# Sex Differences in Long-Term Mortality and Functional Outcome After Rehabilitation in Patients With Severe Stroke

**DOI:** 10.3389/fneur.2020.00084

**Published:** 2020-02-18

**Authors:** Domenico Scrutinio, Petronilla Battista, Pietro Guida, Bernardo Lanzillo, Rosanna Tortelli

**Affiliations:** ^1^Department of Neurorehabilitation, Istituti Clinici Scientifici Maugeri IRCCS, Pavia, Italy; ^2^Department of Neurodegenerative Disease, University College London (UCL) Institute of Neurology, London, United Kingdom

**Keywords:** sex, stroke, mortality, functional outcome, rehabilitation

## Abstract

**Objective:** We sought to determine sex differences in outcomes in patients with severe stroke who had been admitted to inpatient rehabilitation.

**Methods:** We studied 1,316 patients aged 18 to 99 (mean 72) classified as case-mix groups 0108, 0109, and 0110 of the Medicare case-mix classification system. These groups encompass the most severe strokes. Three outcomes were analyzed: (1) 3-year mortality from admission to rehabilitation; (2) combined outcome of transfer to acute care or death within 90 days from admission to rehabilitation; (3) functional outcome, including proportional recovery in motor functioning and good functional outcome as defined by achievement of a Functional Independence Measure (FIM)-motor score ≥65 points at discharge. Multivariable regression analyses were used to assess sex-difference in each outcome between women and men. The covariates examined included age, marital status, comorbidities, time from stroke onset to rehabilitation admission <30 days, ischemic stroke, dysphagia, neglect, motor FIM score at admission, and cognitive FIM score at admission.

**Results:** Kaplan-Meier estimated 3-year mortality rate was 20.7% in women and 22.0% in men. The crude hazard ratio (HR) of death for women compared with men was 0.94 (95% CI 0.74–1.20). After adjustment for significant covariates, the HR of 3-year mortality was 0.73 (95% CIs 0.56–0.96; *p* = 0.025). Comorbidity, including diabetes, anemia, coronary artery disease, atrial fibrillation, and chronic obstructive pulmonary disease, significantly increased mortality risk by 49–88%. The incidence of the combined outcome was 8.3% in women and 8.4% in men. The crude HR of the combined end-point for women compared with men was 1.05 (95% CI 0.72–1.53). After adjustment for significant covariates, the HR was 0.95 (95% CIs 0.65–1.40; *p* = 0.810). Likewise, no significant difference in proportional recovery or in the rate of achievement of a good functional outcome between women and men was observed.

**Conclusion:** Among patients admitted to inpatient rehabilitation after severe stroke, women and men had comparable crude mortality rates at 3 years. After multivariable adjustment, however, women had lower mortality risk. No sex-differences in the risk of being transferred to acute care or dying within 90 days from admission to rehabilitation or in responsiveness to rehabilitation were observed.

## Introduction

Stroke is a leading cause of death and disability worldwide (1). According to the most recent report from the Global Burden of Disease Stroke Collaboration, there were 1·03 million incident strokes in Western Europe and 0·81 in North America in 2016 (1). Despite a substantial decline in stroke mortality in recent decades, stroke is the second leading cause of cardiovascular death worldwide (2). Approximately 20–25% of stroke survivors present severe disability (3). Comorbidity is prevalent in stroke patients and affects both life expectancy and disability (4).

Understanding sex differences in epidemiology, pathophysiology, outcomes, and treatment effectiveness is important since could provide evidence for reducing potential sex disparities. Previous studies of sex differences in post-stroke outcomes provided conflicting findings (5). Two recently published systematic reviews from the International Stroke Outcomes Study (INSTRUCT) research group suggest that sex differences in mortality and functional outcomes are eliminated after adjustment for age, pre-stroke functional limitation, and stroke severity (6, 7). The higher mortality risk for women was even reversed after adjustment (6). However, as noted by the Authors, the variability in measures of stroke severity used in individual studies may have introduced some bias in adjusted estimates (6). Furthermore, Gall et al., considering patient-reported outcomes, showed that women had worse functional outcomes than men, which persisted after accounting for a range of covariates (8). However, the role of rehabilitation was yet not addressed in any of the above-mentioned studies. Despite the large number of studies on sex differences in stroke, only few data are available on the relative responsiveness of women and men to rehabilitation (6). Early rehabilitation is effective in fostering functional recovery and may positively affect mortality (9, 10). An association between functional gain achieved with rehabilitation and mortality risk also has been demonstrated (11, 12). Another aspect to consider is that most individual studies have been based on patient populations with prevalent mild or moderate stroke and the relevance of research findings to the critical population with severe stroke remains elusive. Severe stroke is associated with increased burden of mortality and disability, wider interindividual variation in responsiveness to rehabilitation, and higher healthcare and social costs compared with less severe strokes (12, 13). Better understanding of sex differences in this challenging patient population could provide new insightful information and opportunities to reduce potential sex disparities. To address this issue, we studied 1,316 patients classified as case-mix groups (CMGs) 0108, 0109, and 0110 of the Medicare case-mix classification system (14), which was specifically developed to account for “the level of severity of a given case” (15). Case-mix groups 0108, 0109, and 0110 encompass the most severe strokes.

## Materials and Methods

### Participants

**Figure 1:**
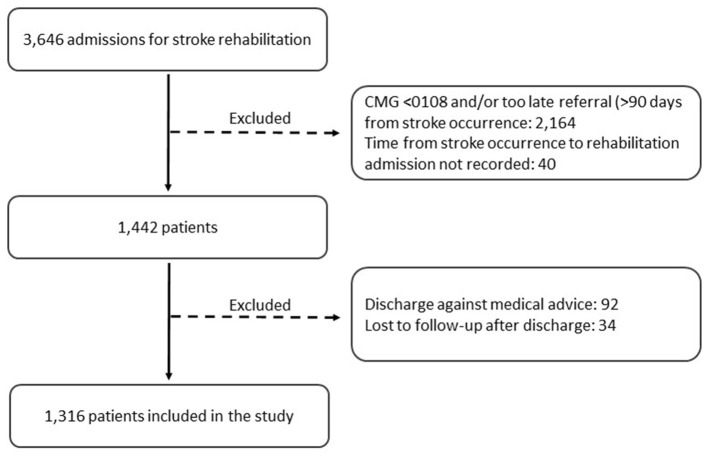
Flowchart of patient selection.

Patients were recruited from the specialized stroke rehabilitation units of the Maugeri inpatient rehabilitation facilities (IRFs) of Cassano Murge (Bari), Telese Terme (Benevento), and Montescano (Pavia) in Italy. Enrolment periods varied among the participating centers but ran from February 2002 to September 2016 overall. A total of 3,646 patients admitted for stroke rehabilitation were identified using a computer-generated list obtained from our administrative database and by reviewing electronic medical records. We included patients admitted to stroke rehabilitation units ≤90 days from stroke occurrence and classified as CMG 0108 (weighted Functional Independence Measure [wFIM] motor score <26.15 and age >84.5), 0109 (wFIM motor score >22.35 and <26.15, and age <84.5), or 0110 (wFIM motor score <22.35 and age <84.5) of the Medicare case-mix classification system (14). Patients classified as CMGs 0101 to 0107, admitted to rehabilitation >90 days from stroke occurrence, or discharged against medical advice were excluded. Of the 3,646 patients, 1,316 fulfilling the selection criteria were included in the study. Figure 1 shows the flowchart of patient selection. The Medicare classification system distinguishes 10 CMGs for stroke rehabilitation. Patients are assigned into one of the 10 distinct CMGs, based on age, the sum of weighted ratings for 12 FIM-motor items (transfer to tub or shower item is excluded), and the sum of FIM cognitive ratings (14). The FIM is currently the most widely used measure to describe the degree of impairment in activities of daily living in clinical practice. The motor-FIM score consists of 13 items assessing four domains of function (self-care, sphincter control, transfers, and locomotion). The cognitive-FIM score consists of five items assessing two domains (communication and social cognition). Each item is scored on a 7-point Likert scale, from 1 (total dependence) to 7 (total independence).

The characteristics of the three participating stroke rehabilitation units have been described previously (11, 12). The participating rehabilitation units are certified ISO9001 Quality Management Systems for activities of rehabilitation and share common rehabilitation programs. The interdisciplinary stroke rehabilitation teams comprise the following professionals with expertise in stroke rehabilitation: neurologist, physiatrist, physiotherapist, occupational therapist, speech and language therapist, neuropsychologist, and nurse. Trained therapists recorded admission and discharge FIM scores, as part of our formal rehabilitation program. Individual rehabilitation programs were structured to provide as much scheduled rehabilitation therapy as possible, with the objective of providing therapy for 3 h per day for 5 days and for 1 h for 1 day of each week. Conformity to this standard is subject to periodic external audit by independent auditors of the Regional Health Agencies. The study was approved by our Institutional Review Board. Patients' data were deidentified.

### Data Collection

All data were extracted from the electronic Hospital Information System networked between the participating centers. Vital status was ascertained by linking with the regional Health Information System.

### Definitions

Coronary artery disease (CAD) was diagnosed based on a documented history of myocardial infarction, percutaneous coronary angioplasty, or coronary artery bypass grafting, or a previous hospitalization for CAD. Renal dysfunction was defined as estimated glomerular filtration rate <60 mL/min/1.73 m^2^. Anemia was defined as hemoglobin <12 g/dL in women and <13 g/dL in men. Atrial fibrillation (AF) was diagnosed based on admission electrocardiogram. Chronic obstructive pulmonary disease (COPD) was diagnosed based on patient's medical records documenting a past diagnosis of COPD, chronic medication use for COPD, and/or previous hospitalizations for exacerbation of COPD. The Bedside Swallowing Assessment Scale, administered by a trained speech therapist, was used to diagnose dysphagia. If concerns regarding the safety and efficiency of swallow function emerged from the scale, a fiberoptic endoscopic evaluation of swallowing was performed. The Semi-Structured Scale for the Functional Evaluation of Hemi-inattention was used to diagnose personal neglect.

### Outcomes

The following clinical and functional outcomes were analyzed: (1) all-cause mortality up to 3 years from admission to rehabilitation; (2) combined outcome of transfer to acute care or death within 90 days from admission to rehabilitation, whatever came first; (3) functional outcome. Two measures of functional outcome were used: (1) proportional recovery in motor functioning, as expressed by motor-FIM effectiveness, and (2) good functional outcome as defined by achievement of a FIM-motor score ≥65 points at discharge. Proportional recovery in motor functioning is calculated by the formula: (discharge motor-FIM score–admission motor-FIM score)/(maximum motor-FIM score–admission motor-FIM score) × 100 (16). Proportional recovery expresses the achieved proportion of available improvement in motor functioning (16). According to Stinear, “measuring proportional recovery enables the detection of treatment effects despite interindividual variability in absolute recoveries and outcomes” (17). To facilitate the interpretation of functional improvement, we also calculated the proportion of women and men who achieved a good functional outcome as defined by FIM-motor score ≥65 points at discharge. Based on Rasch analysis, patients with a score ≥65 “usually require either supervision or minimal assistance with mobility and self-care, indicating that the patient' physical care requirements for daily activities are minimal” (18).

### Statistical Analysis

Data are reported as mean and standard deviation (SD) or median and interquartile range (IQR) for continuous variables or percentage for categorical variables. No variable was missing more than 0.1% of values. Rates of mortality were estimated by means of the Kaplan-Meier method and were compared between women and men using the log-rank test.

#### Covariates

The covariates examined included age (per 5-year increase above 65), marital status (married/not married), hypertension, diabetes, COPD, history of CAD, AF, anemia, renal dysfunction (estimated glomerular filtration rate <60 mL/min/1.73 m2), time from stroke onset to rehabilitation admission <30 days, ischemic stroke, dysphagia, neglect, motor FIM score at admission, and cognitive FIM score at admission. These variables were selected based on availability at time of presentation and prior studies showing an association with the outcomes of interest (4, 6, 11, 12, 17, 19–32) and were included in all analyses.

#### Three-Year All-Cause Mortality

Crude hazard ratio (HR) of death for women compared with men was estimated by univariable Cox regression analysis. Adjusted HR was estimated by multivariable backward stepwise Cox regression analysis (*p* > 0.05 to remove). Schoenfeld residuals after fitting Cox model were evaluated to test proportional-hazards assumption. Interactions between sex and covariates were estimated by using the likelihood ratio test.

In addition, since unmeasured potential confounding factors may affect hazard estimates, a sensitivity analysis was performed to explore the potential confounding effect of an unknown or unmeasured variable on the association of sex with 3-year survival (33). Hazard ratios for women vs. men adjusted for a hypothetical unmeasured binary variable with different distribution in the two sexes were estimated. The effect was quantified assuming a HR of 1.3, 1.4, and 1.5.

To explore the association between comorbidities and mortality, recursive-partitioning analysis (for censored survival data) was applied to cluster patients into risk subgroups according to comorbidities and to identify the combinations of comorbidities that were most influential for 3-year mortality, adjusting for age, sex, and type of stroke (ischemic or hemorrhagic) (34).

#### Combined Outcome

Crude and adjusted HRs of the combined outcome of transfer to acute care or death within 90 days from admission to rehabilitation for women compared with men were estimated as described above.

#### Functional Outcome

The association of baseline covariates with proportional recovery in motor functioning was assessed using beta regression. A multivariable analysis was performed to model the proportion of recovery on the basis of significant covariates and to estimate the effect of sex. Beta coefficients with standard error (SE) were reported. Crude odds ratio (OR) of good functional outcome for women compared with men was estimated by univariable logistic regression model. Adjusted OR was estimated by multivariable logistic regression analysis. These analyses were limited to the 1,209 patients who completed rehabilitation.

Sex, as main exposure variable, was included into all multivariable models regardless of significance level.

Finally, for each outcome, a full adjusted analysis was performed including all covariates.

All analyses were conducted using STATA software, version 14 (Stata-Corp LP, College Station, Tex).

## Results

Of the 1,316 patients included in the study, 587 (44.6%) were women and 729 (55.4%) men. Table 1 shows baseline characteristics stratified by sex.

**Table 1:** Baseline characteristics stratified by sex.

	**All (*N* = 1,316)**	**Women (*N* = 587)**	**Men (*N* = 729)**
**Demographics**			
Age (years), mean (SD)	72 (12)	73 (11)	71 (11)
<65 years, *n* (%)	320 (24.3)	111 (18.9)	209 (28.7)
65 to 74 years, *n* (%)	376 (28.6)	164 (27.9)	212 (29.1)
≥75 years, *n* (%)	620 (47.1)	312 (53.2)	308 (42.2)
Marital status–married, *n* (%)	941 (71.5)	342 (58.3)	599 (82.2)
**Comorbidities**			
Hypertension, *n* (%)	954 (72.5)	441 (75.3)	513 (70.4)
Diabetes, *n* (%)	393 (29.9)	170 (29.0)	223 (30.6)
COPD, *n* (%)	189 (14.4)	80 (13.7)	109 (15.0)
CAD, *n* (%)	168 (12.8)	57 (9.7)	111 (15.2)
Atrial fibrillation, *n* (%)	344 (26.2)	203 (34.6)	141 (19.3)
Anemia (hemoglobin <13 g/dL in men, <12 g/dL in women), *n* (%)	465 (35.3)	194 (33.1)	271 (37.2)
Renal dysfunction (eGFR <60 mL/min/1.73 m^2^), *n* (%)	233 (17.7)	118 (20.1)	115 (15.8)
**Stroke-related characteristics**			
CMG 108, *n* (%)	153 (11.6)	75 (12.8)	78 (10.7)
CMG 109, *n* (%)	123 (9.3)	51 (8.7)	72 (9.9)
CMG 110, *n* (%)	1,040 (79.0)	461 (78.5)	579 (79.4)
Time from stroke onset to rehabilitation admission (days), median (IQR)	23.7 (16.6)	23.2 (16.3)	24.1 (16.9)
Time from stroke onset to rehabilitation admission ≤30 days, *n* (%)	993 (75.5)	449 (76.5)	544 (74.6)
Ischemic stroke, *n* (%)	1,051 (79.9)	488 (83.1)	563 (77.2)
Hemorrhagic stroke, *n* (%)	265 (20.1)	99 (16.9)	166 (22.8)
Dysphagia, *n* (%)	277 (21.0)	120 (20.4)	157 (21.5)
Neglect, *n* (%)	187 (14.2)	87 (14.8)	100 (13.7)
Aphasia, *n* (%)	581 (44.1)	255 (43.4)	326 (44.7)
**Site of impairment**			
Right body, *n* (%)	663 (50.4)	291 (49.6)	372 (51.0)
Left body, *n* (%)	653 (49.5)	296 (50.4)	357 (49.0)
12-item motor-FIM score at admission, mean (SD)	17.4 (5.6)	17.3 (5.6)	17.4 (5.5)
Cognitive-FIM score at admission, mean (SD)	16.7 (9.3)	16.5 (9.3)	16.8 (9.2)
Length of stay (days), mean (SD)	54 (17)	54 (16)	54 (18)
**Laboratory findings**			
Blood urea nitrogen (mg/dl), mean (SD)	21 (11)	20 (11)	22 (10)
Serum creatinine (mg/dl), mean (SD)	0.89 (0.35)	0.79 (0.32)	0.96 (0.36)
eGFR (mL/min/1.73 m^2^), mean (SD)	83 (26)	80 (25)	86 (26)
Serum sodium (mmol/l), mean (SD)	140.6 (4.1)	141.0 (4.0)	140.3 (4.1)
Serum sodium <135 mmol/l, *n* (%)	56 (4.2)	19 (3.2)	37 (5.1)
Hemoglobin (g/dl), mean (SD)	13.1 (1.8)	12.6 (1.6)	13.5 (1.8)
Total cholesterol (mg/dl), mean (SD)	163 (43)	176 (45)	153 (39)

*N, denotes number; SD, standard deviation; COPD, chronic obstructive pulmonary disease; CAD, coronary artery disease; CMG, case-mix group; eGFR, estimated glomerular filtration rate*.

### Three-Year Mortality

**Figure 2:**
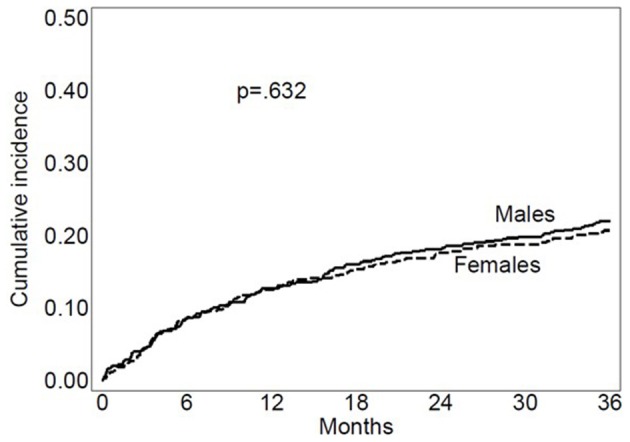
Kaplan Meier mortality curves for women and men.

A total of 3,141 person-years of follow-up were examined during which 269 deaths (8.6 deaths/100 person-years) occurred. Median follow-up was 1,095 (IQR 668-1095) days. 79.3% of the survivors had a complete 3-year follow-up. Kaplan-Meier estimated 3-year mortality rate was 20.7% in women and 22.0% in men (Figure 2). Besides sex, age, marital status, diabetes, CAD, COPD, AF, anemia, dysphagia, neglect, and cognitive status were significantly associated with mortality risk at multivariable Cox analysis (Supplementary Table 1). There was no significant interaction between sex and any covariate with regard to 3-year mortality. Table 2 shows crude and multivariable-adjusted HR of mortality for women compared with men. Female sex was associated with significantly decreased hazard for mortality compared with male sex. Estimates for sex remained virtually unchanged in fully adjusted models, including all covariates (Table 2).

**Table 2:** Crude and multivariable-adjusted estimates of the association of female sex with mortality and the combined outcome.

	***N***	**Crude HR (95% CI)**	***p*-value**	**Adjusted HR (95% CI)**	***p*-value**	**Fully adjusted HR (95% CI)**	***p*-value**
Three-year mortality	1,316	0.94 (0.74–1.20)	0.632	0.73 (0.56–0.96)^*^	0.025	0.73 (0.56–0.95)	0.022
Combined outcome	1,316	1.05 (0.72–1.53)	0.789	0.95 (0.65–1.40)^**^	0.810	0.94 (0.62–0.96)	0.770

N, denotes the number of patients; HR, hazard ratio; CI, confidence interval.

* Adjusted for age, marital status, diabetes, coronary artery disease, chronic obstructive pulmonary disease, atrial fibrillation, anemia, dysphagia, neglect, and cognitive FIM score.

** *Adjusted for atrial fibrillation, dysphagia, anemia, and low cognitive FIM score*.

Sensitivity analysis showed that hazard estimate of 3-year mortality may be sensitive to unknown or unmeasured confounders. Supplementary Table 2 shows HRs of 3-year mortality for women vs. men adjusted for a hypothetical unknown or unmeasured binary variable. As an example, an unmeasured binary confounder with a HR of 1.4 and a prevalence of 40% in men and 20% in women would raise the upper confidence limit of HR beyond 1.00.

**Figure 3:**
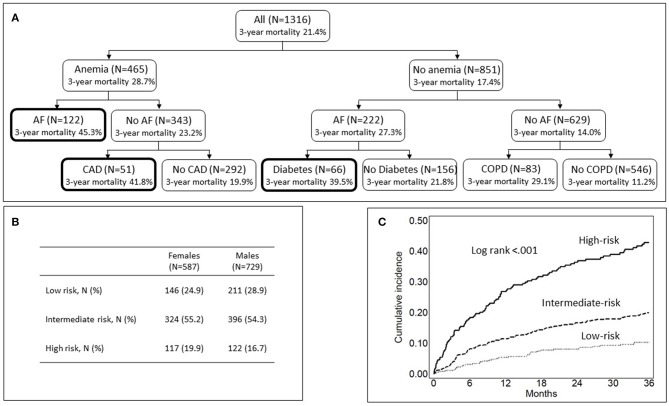
Classification of patients into risk-of-death categories based on comorbidity. **(A)** Shows the results of recursive partitioning analysis. Bold lines denote the high-risk category. **(B)** Shows the distribution of females and males across the three risk categories. **(C)** Shows Kaplan Meier mortality curves for the high-, intermediate-, and low-risk categories.

Figure 3A depicts the results of recursive-partitioning analysis. The three highest risk subgroups included patients with concurrent anemia and AF (3-year mortality rate: 45.3%), anemia and CAD (3-year mortality rate: 41.8%), or atrial fibrillation and diabetes (3-year mortality rate: 39.5%). Overall, 239 (18.2%) patients were at high risk of death because of the combination of these comorbidities. These patients were grouped into a single high-risk category. The 357 (27.1%) patients without any comorbidity among diabetes, anemia, CAD, AF, and COPD were grouped into the low-risk category. The remaining 720 patients (54.7%) were grouped into an intermediate-risk category. There was no difference in the distribution of females and males across the three risk categories (*p* = 0.145) (Figure 3B). In comparison with the low-risk group, the adjusted HR of 3-year mortality for the high-risk category was 3.93 (95% CIs 2.64–5.84) and that for the intermediate-risk category 1.83 (95% CIs 1.26–2.66), regardless of age, sex, and type of stroke (Supplementary Table 3). Figure 3C shows Kaplan-Meier mortality curves for high-, intermediate- and low-risk categories.

### Combined Outcome

The incidence of the combined outcome was 8.3% in women and 8.4% in men. At multivariable analysis, atrial fibrillation, dysphagia, anemia, and low cognitive FIM score were significantly associated with increased risk of the combined outcome (Supplementary Table 4). Table 2 shows crude and multivariable-adjusted HR for the combined outcome for women compared with men. Since sex was not retained in multivariable analysis, it was forced into the multivariable model. After adjustment for significant covariates, female sex was not associated with risk of the combined outcome compared with male sex. Estimates for sex remained virtually unchanged in fully adjusted models, including all covariates (Table 2).

### Functional Outcome

Mean proportional recovery in motor functioning achieved in women was statistically significantly lower than that achieved in men (31.4 ± 25.7 percent vs. 35.0 ± 24.8 percent; *p* = 0.014). At multivariable analysis, age, marital status, AF, time to rehabilitation admission from stroke onset <30 days, ischemic stroke, dysphagia, neglect, and admission motor- and cognitive-FIM scores were associated with proportional recovery (Supplementary Table 5). Since sex was not retained in multivariable analysis, it was forced into the multivariable model. After adjustment for significant covariates, no difference in proportional recovery between women and men was found (Table 3). The proportion of women and men who achieved a good functional outcome, defined by a FIM-motor score ≥65 points at discharge, was 15.4 and 16.4%, respectively. At multivariable logistic regression analysis, age, time to rehabilitation admission <30 days, ischemic stroke, dysphagia, neglect, motor and cognitive FIM scores were significantly associated with good functional outcome (Supplementary Table 6). Table 3 shows crude and multivariable-adjusted HR for the combined outcome for women compared with men. Since sex was not retained in multivariable analysis, it was forced into the multivariable model. After adjustment for significant covariates, female sex was not associated with odds of good functional outcome. Estimates for sex remained virtually unchanged in fully adjusted models, including all covariates.

**Table 3:** Crude and multivariable-adjusted estimates of the association of female sex with functional outcome.

	***N***	**Crude β regression coefficient (SE)**	***p*-value**	**Adjusted β regression coefficient (SE)**	***p*-value**	**Fully adjusted β regression coefficient (SE)**	***p*-value**
Proportional recovery	1,209	−0.189 (0.059)	0.002	−0.035 (0.056) ^*^	0.534	−0.045 (0.056)	0.422
		**Crude OR** **(95% CI)**		**Adjusted OR** **(95% CI)**		**Fully adjusted OR** **(95% CI)**	
Good functional outcome	1,209	0.92 (0.68–1.26)	0.613	1.11 (0.78–1.57) ^**^	0.569	1.20 (0.84–1.72)	0.317

N, denotes the number of patients; SE, standard error; OR, odds ratio.

* Adjusted for age, marital status, atrial fibrillation, time to rehabilitation admission from stroke onset <30 days, ischemic stroke, dysphagia, neglect, and admission motor- and cognitive-FIM scores significant covariates.

** *Adjusted for age, time to rehabilitation admission <30 days, ischemic stroke, dysphagia, neglect, motor and cognitive FIM scores*.

## Discussion

We investigated sex differences in outcomes in a large patient cohort with severe stroke who had been admitted to inpatient rehabilitation. There are three major findings of this study: (1) women in comparison with men were associated with a 27% lower adjusted 3-year risk of death. (2) Comorbidity had a statistically and clinically significant impact on mortality, regardless of age, sex and type of stroke. (3) No sex difference in the incidence of the combined outcome or in responsiveness to rehabilitation was observed.

Women and men had comparable crude mortality rates at 3 years. However, women had a 27% lower adjusted 3-year risk of death compared with men, possibly reflecting the female survival advantage until late in life in the general population (35). Female sex remained significantly associated with lower risk of death even after full adjustment. As discussed by Austed (35), mechanistic hypotheses to explain the female survival advantage focus on hormones, oxidative damage to DNA, and asymmetric inheritance of sex chromosomes. The finding of a lower adjusted mortality risk for women vs. men is consistent with the meta-analysis of Phan et al. (6), where a statistically significant 24% lower adjusted mortality rate ratio at 5 years for women compared with men was estimated. Our finding is also in line with the study of Bots et al. showing that mortality rate after stroke is higher among men than women across age groups until old age (19).

It has been suggested that stroke severity dominates risk for poor outcome in patients with severe stroke (36). Our data indicate that, in this critical subset of patients, comorbidities are significantly associated poor long-term survival. Consistent with previous studies (20–22), diabetes, CAD, COPD, AF, and anemia were independently associated with increased mortality, regardless of age, sex, and type of stroke. In comparison with the patient subgroup without any of these comorbidities, the high-risk subgroup had a nearly four-fold time increased risk of death within 3 years. Atrial fibrillation and anemia also doubled the risk of transfer to acute care and death within 90 days from rehabilitation admission. These findings are of particular interest because comorbidities are amenable to interventions. Recently, the American Stroke Association recommended that the focus of post-acute care should be on maximizing recovery, reducing mortality, and preventing recurrent strokes and cardiovascular events (37). Reasonably, tailoring management and secondary prevention according to comorbidities would result in better outcomes. Because of insufficient evidence, however, guidelines fail to provide guidance for care of stroke patients with comorbidity (38). Further research addressing care of patients with comorbidity is needed.

No differences in the incidence of the combined outcome of transfer from the rehabilitation setting to acute care or 90-day case fatality between women and men was observed. Likewise, the extent of functional recovery did not differ between women and men, even after multivariable adjustment. It should however be noted that a large proportion of interindividual variability in functional outcome remains unexplained. In a retrospective analysis of the Uniform Data System for Medical Rehabilitation data set, the proportion of functional recovery explained by a predictive model incorporating age, admission FIM motor score, and walking distance was as low as 10.7% (23). Identifying stroke recovery biomarkers could allow enhancing the ability to explain interindividual differences in post-stroke outcomes. Two recent meta-analyses showed that genetic variants and the severity of white matter hyperintensities, as assessed by magnetic resonance imaging or computed tomography at the time of stroke, are associated with functional outcome after ischemic stroke (39, 40). In another study, a panel of five biomarkers covering distinct pathophysiological pathways provided incremental prognostic information beyond that provided by a clinical model in predicting major disability and mortality after stroke (41).

Taken together, our data are in line with the sex mortality-morbidity paradox that women have lower mortality rates from most causes of death, but more years lived with disability (35).

The terms sex and gender have often been used interchangeably in studies that investigated differences in disease outcomes between men and women. However, sex and gender are conceptually distinct. While sex refers to biological and physiological characteristics, gender refers to “psychological, social, and cultural factors that shape attitudes, behaviors, and knowledge” (42). Sex and gender are both important determinants of health and response to interventions (42, 43). Thus, integrating sex- and gender-based analysis can lead to improved research methodology and improved assessment of differences in disease outcomes (42, 43). As an example, using a binary gender index (masculinity vs. femininity), Pelletier et al. found that feminine traits of personality were associated with adverse outcomes in young patients with acute coronary syndromes, regardless of sex (44). Potential pathways by which gender might affect post-stroke rehabilitation outcomes include social isolation, socioeconomic status, education, marital status, poorer pre-stroke function, level of anxiety, depression, and interaction with rehabilitation team and the doctor. Because of the complex and multidimensional nature of gender and the lack of standardized methods of analysis, however, operationalizing the intersection of gender and sex into scientific research remains a very challenging task (42, 43).

### Limitations

Our study has strengths and limitations. To our knowledge, this is the first study specifically addressing sex differences in the critical population of patients with severe stroke. This study adds to previous knowledge by highlighting the impact of comorbidity on long-term mortality and by showing the absence of sex differences in responsiveness to rehabilitation in patients with severe stroke admitted to post-acute rehabilitation. Several limitations should be mentioned. We used hospital-based data that may be prone to selection bias. However, although patients with severe stroke are less likely to be referred to inpatient rehabilitation facilities than those with mild/moderate stroke, access to rehabilitation is similar for women and men (6, 45). Since women with stroke are in general older than men and oldest old patients are less likely to undergo inpatient rehabilitation, an age-related selection bias may have occurred in our study. We used a stepwise approach based on statistical significance to select significant covariates. As reviewed by Talbot and Massamba (46), stepwise methods may overestimate exposure effects and underestimate statistical uncertainty. However, as recommended by Talbot and Massamba (46), we also reported the results from the fully adjusted models. Estimates for sex remained virtually unchanged in fully adjusted models. The retrospective design of the study did not allow accounting for other possible confounders not recorded in our data set. Sensitivity analysis showed that hazard estimates might be sensitive to unknown or unmeasured confounders, such as premorbid functional status. Poor pre-stroke functional status is more prevalent among women than in men and has generally been recognized as a predictor of worse outcomes in stroke survivors. In the meta-analysis of Phan et al., partial adjustment for pre-stroke disability alone attenuated the adverse effect of female sex on 5-year mortality by 55% (6). Thus, it is likely that adjustment for pre-stroke disability in our study would have resulted in even lower adjusted mortality risk for women compared with men. Moreover, some of the included covariates in the multivariable models could be intermediates on the causal pathway between exposure and outcomes, rather than confounders. As noted by Schisterman et al. (47), with adjustment for an intermediate variable in multivariable modeling, the observed association between the exposure and outcome will be a null-biased estimate of the total causal effect. This limitation should be taken into account in the interpretation of our findings. We did not examine the prognostic role of neuroimaging, which could provide incremental prognostic information over clinical and functional variables (40). Finally, we could not assess the causes of death. However, death certificates may lack accuracy (48).

## Conclusion

In conclusion, women and men had comparable crude mortality rates at 3 years. After multivariable adjustment, however, women had lower mortality risk, probably reflecting the higher longevity of women. Comorbidity significantly affected the likelihood of survival, regardless of age, sex and type of stroke. No sex-differences in the risk of being transferred to acute care or dying within 90 days from admission to rehabilitation or in responsiveness to rehabilitation were observed.

## Data Availability Statement

The datasets generated for this study can be found upon request to the corresponding author of the article.

## Ethics Statement

Ethical review and approval was not required for the study on human participants in accordance with the local legislation and institutional requirements. Written informed consent for participation was not required for this study in accordance with the national legislation and the institutional requirements.

## Author Contributions

DS designed the study, interpreted the data, drafted the initial manuscript and revised the manuscript. PB drafted the initial manuscript and revised the manuscript. PG conducted data analysis, prepared the figure, and revised the manuscript. BL interpreted the data and revised the manuscript. RT designed the study, interpreted the data, and revised the manuscript. All authors have contributed to manuscript revision, read, and approved the submitted version.

### Conflict of Interest

The authors declare that the research was conducted in the absence of any commercial or financial relationships that could be construed as a potential conflict of interest.
