# Supplementary material for: Sex Differences in Long-Term Mortality and Functional Outcome After Rehabilitation in Patients With Severe Stroke
Source: Front Neurol. 2020 Feb 18;11:84. doi: 10.3389/fneur.2020.00084 (PMC7040356; doi:10.3389/fneur.2020.00084)
Supplement: Supplementary file 1 [file Table_1.DOCX]

Supplementary Table I. Multivariable Cox regression analysis for 3-year mortality.

| Covariate | Adjusted HR  (95% CI) | p value |
| --- | --- | --- |
| Age (per 5-year increase above 65) | 1.30 (1.20-1.42) | <.001 |
| Marital status - married | 0.66 (0.50-0.87) | .003 |
| Diabetes | 1.58 (1.23-2.03) | <.001 |
| COPD | 1.49 (1.12-1.99) | .007 |
| CAD | 1.88 (1.38-2.56) | <.001 |
| Atrial fibrillation | 1.62 (1.25-2.09) | <.001 |
| Anemia | 1.51 (1.18-1.92) | .001 |
| Dysphagia | 1.49 (1.15-1.94) | .003 |
| Neglect | 1.48 (1.06-2.06) | .020 |
| Cognitive FIM score (per 5-unit increase) | 0.77 (0.72-0.84) | <.001 |

Abbreviations. HR denotes hazard ratio, CI confidence intervals, COPD chronic obstructive pulmonary disease, CAD coronary artery disease.

Supplementary table II. Hazard ratios and 95% confidence intervals of 3-year mortality for women vs men for an unknown or unmeasured binary confounder with a hazard ratio of 1.3, 1.4, or 1.5.

| Confounder with hazard ratio for death of 1.3 | | | | | | | | | | |
| --- | --- | --- | --- | --- | --- | --- | --- | --- | --- | --- |
|  | **Females** | | | | | | | | | |
| **Males** | **0.0** | **0.1** | **0.2** | **0.3** | **0.4** | **0.5** | **0.6** | **0.7** | **0.8** | **0.9** |
| **0.0** | **0.73 (0.56-0.96)** |  |  |  |  |  |  |  |  |  |
| **0.1** | **0.76 (0.58-0.99)** |  |  |  |  |  |  |  |  |  |
| **0.2** | 0.78 (0.59-1.02) | **0.76 (0.58-0.99**) |  |  |  |  |  |  |  |  |
| **0.3** | 0.80 (0.61-1.05) | 0.78 (0.59-1.02) | **0.75 (0.58-0.99)** |  |  |  |  |  |  |  |
| **0.4** | 0.82 (0.63-1.08) | 0.80 (0.61-1.05) | 0.78 (0.59-1.02) | **0.75 (0.58-0.99)** |  |  |  |  |  |  |
| **0.5** | 0.84 (0.64-1.11) | 0.82 (0.63-1.07) | 0.80 (0.61-1.04) | 0.77 (0.59-1.01) | **0.75 (0.58-0.99)** |  |  |  |  |  |
| **0.6** | 0.87 (0.66-1.13) | 0.84 (0.64-1.10) | 0.82 (0.62-1.07) | 0.79 (0.61-1.04) | 0.77 (0.59-1.01) | **0.75 (0.58-0.99)** |  |  |  |  |
| **0.7** | 0.89 (0.68-1.16) | 0.86 (0.66-1.13) | 0.84 (0.64-1.10) | 0.82 (0.62-1.07) | 0.79 (0.61-1.04) | 0.77 (0.59-1.01) | **0.75 (0.58-0.99)** |  |  |  |
| **0.8** | 0.91 (0.70-1.19) | 0.88 (0.68-1.16) | 0.86 (0.66-1.12) | 0.84 (0.64-1.09) | 0.81 (0.62-1.06) | 0.79 (0.60-1.04) | 0.77 (0.59-1.01) | **0.75 (0.57-0.99)** |  |  |
| **0.9** | 0.93 (0.71-1.22) | 0.91 (0.69-1.19) | 0.88 (0.67-1.15) | 0.86 (0.65-1.12) | 0.83 (0.64-1.09) | 0.81 (0.62-1.06) | 0.79 (0.60-1.03) | 0.77 (0.59-1.01) | **0.75 (0.57-0.98)** |  |
| **1.0** | 0.95 (0.73-1.25) | 0.93 (0.71-1.21) | 0.9 (0.69-1.18) | 0.88 (0.67-1.15) | 0.85 (0.65-1.12) | 0.83 (0.63-1.09) | 0.81 (0.62-1.06) | 0.79 (0.6-1.03) | 0.77 (0.59-1.01) | **0.75 (0.57-0.98)** |

| Confounder with hazard ratio for death of 1.4. | | | | | | | | | | |
| --- | --- | --- | --- | --- | --- | --- | --- | --- | --- | --- |
|  | **Females** | | | | | | | | | |
| **Males** | **0.0** | **0.1** | **0.2** | **0.3** | **0.4** | **0.5** | **0.6** | **0.7** | **0.8** | **0.9** |
| **0.0** | **0.73 (0.56-0.96)** |  |  |  |  |  |  |  |  |  |
| **0.1** | **0.76 (0.58-1.00)** |  |  |  |  |  |  |  |  |  |
| **0.2** | 0.79 (0.61-1.04) | **0.76 (0.58-1.00)** |  |  |  |  |  |  |  |  |
| **0.3** | 0.82 (0.63-1.08) | 0.79 (0.60-1.04) | **0.76 (0.58-1.00)** |  |  |  |  |  |  |  |
| **0.4** | 0.85 (0.65-1.11) | 0.82 (0.63-1.07) | 0.79 (0.60-1.03) | **0.76 (0.58-1.00)** |  |  |  |  |  |  |
| **0.5** | 0.88 (0.67-1.15) | 0.85 (0.65-1.11) | 0.82 (0.62-1.07) | 0.79 (0.60-1.03) | **0.76 (0.58-0.99)** |  |  |  |  |  |
| **0.6** | 0.91 (0.7-1.19) | 0.88 (0.67-1.15) | 0.84 (0.64-1.10) | 0.81 (0.62-1.06) | 0.78 (0.60-1.03) | **0.76 (0.58-0.99)** |  |  |  |  |
| **0.7** | 0.94 (0.72-1.23) | 0.90 (0.69-1.18) | 0.87 (0.66-1.14) | 0.84 (0.64-1.10) | 0.81 (0.62-1.06) | 0.78 (0.60-1.03) | **0.76 (0.58-0.99)** |  |  |  |
| **0.8** | 0.97 (0.74-1.27) | 0.93 (0.71-1.22) | 0.90 (0.69-1.17) | 0.87 (0.66-1.13) | 0.84 (0.64-1.09) | 0.81 (0.62-1.06) | 0.78 (0.60-1.02) | **0.76 (0.58-0.99)** |  |  |
| **0.9** | 1.00 (0.76-1.31) | 0.96 (0.73-1.26) | 0.92 (0.71-1.21) | 0.89 (0.68-1.17) | 0.86 (0.66-1.13) | 0.83 (0.64-1.09) | 0.81 (0.62-1.05) | 0.78 (0.60-1.02) | **0.76 (0.58-0.99)** |  |
| **1.0** | 1.03 (0.79-1.35) | 0.99 (0.75-1.29) | 0.95 (0.73-1.25) | 0.92 (0.70-1.20) | 0.89 (0.68-1.16) | 0.86 (0.65-1.12) | 0.83 (0.63-1.09) | 0.80 (0.61-1.05) | 0.78 (0.59-1.02) | **0.76 (0.58-0.99)** |

Supplementary table III. Hazard ratios for risk categories adjusted for age, sex, type of stroke.

|  | Hazard ratio (95% CIs) | p value |
| --- | --- | --- |
| Age above 65 years (per 5-year increase above 65) | 1.31 (1.21-1.42) | <.001 |
| Female sex | 0.88 (0.62-1.25) | 0.476 |
| Ischemic stroke | 1.12 (0.79-1.59) | 0.521 |
|  |  |  |
| Low-risk category | Reference |  |
| Intermediate-risk category | 1.83 (1.26-2.66) | .001 |
| High-risk group | 3.93 (2.64-5.84) | <.001 |

Supplementary Table IV. Multivariable Cox regression analysis for the combined end-point.

| Covariate | Adjusted HR (95% CI) | p value |
| --- | --- | --- |
| Atrial fibrillation | 2.25 (1.53-3.31) | <.001 |
| Anemia | 2.00 (1.37-2.92) | <.001 |
| Dysphagia | 2.95 (1.99-4.35) | <.001 |
| Cognitive FIM score (per 5-point increase) | 0.81 (0.72-0.92) | .001 |

Abbreviations. HR denotes hazard ratio, CI confidence intervals.

Supplementary Table V. Multivariable Beta regression analysis for proportional recovery.

| Covariate | β Coefficient (SE) | p value |
| --- | --- | --- |
| Age (per 5-year increase above 65) | -0.130 (0.018) | <.001 |
| Marital status - married | 0.209 (0.062) | .001 |
| Atrial fibrillation | -0.212 (0.066) | .001 |
| Time to rehabilitation admission <30 days | 0.337 (0.063) | <.001 |
| Ischemic stroke | -0.219 (0.068) | .001 |
| Dysphagia | -0.312 (0.072) | <.001 |
| Neglect | -0.411 (0.080) | <.001 |
| Motor FIM score (per 5-point increase) | 0.246 (0.028) | <.001 |
| Cognitive FIM score (per 5-point increase) | 0.111 (0.017) | <.001 |

Abbreviations. SE denotes standard error.

Supplementary Table VI. Multivariable logistic regression analysis for good functional outcome.

| Covariate | Adjusted OR  (95% CI) | p value |
| --- | --- | --- |
| Age (per 5-year increase above 65) | 0.75 (0.67-0.85) | <.001 |
| Time to rehabilitation admission <30 days | 1.75 (1.13-2.71) | .012 |
| Ischemic stroke | 0.60 (0.40-0.91) | .015 |
| Dysphagia | 0.44 (0.23-0.83) | .012 |
| Neglect | 0.30 (0.16-0.57) | <.001 |
| Motor FIM score (per 5-points increase) | 1.69 (1.44-1.98) | <.001 |
| Cognitive FIM score (per 5-points increase) | 1.21 (1.10-1.35) | <.001 |

Abbreviations. OR denotes odds ratio, CI confidence intervals.
